# Diagnosis of Pneumonia in Children with Dehydrating Diarrhoea

**Published:** 2014-03

**Authors:** Debasish Saha, Anne Ronan, Wasif Ali Khan, Mohammed Abdus Salam

**Affiliations:** ^1^icddr,b, GPO Box 128, Dhaka 1000, Bangladesh; ^2^Centre for International Health, Department of Preventive and Social Medicine, Dunedin School of Medicine, University of Otago, Dunedin, New Zealand; ^3^Hunter New England Health, University of Newcastle, New South Wales, Australia

**Keywords:** Children, Diagnosis, Diarrhoea, Pneumonia

## Abstract

The World Health Organization (WHO) guidelines for diagnosis of pneumonia are based on the history of cough or difficult breathing and age-adjusted respiration rates. Metabolic acidosis associated with dehydrating diarrhoea also influences the respiration rate. Two hundred and four children, aged 2 to 59 months, with dehydrating diarrhoea and a history of cough and/or fast breathing, were enrolled in a prospective study. Pneumonia diagnoses were made on enrollment and again 6 hours post-enrollment (after initial rehydration), using the WHO guidelines. These were compared with investigators’ clinical diagnosis based on history and findings of physical examination and a chest x-ray at the same time points. Using the WHO guidelines, 149/152 (98%) infants in the 2-11 months age-group and 38/40 (95%) children in the 12-59 months age-group were diagnosed to have pneumonia on enrollment, which dropped to 107 (70%) and 30 (75%) respectively at 6 hours post-enrollment. The specificity of the WHO guidelines for diagnosis of pneumonia was very low (6.9%) at enrollment but increased to 65.5% at 6 hours post-enrollment, after initial rehydration. The specificity of the WHO guidelines for diagnosis of pneumonia in young children is significantly reduced in dehydrating diarrhoea. For young children with dehydrating diarrhoea, rehydration, clinical and radiological assessments are useful in identifying those with true pneumonia.

## INTRODUCTION

In developing countries, 70% of childhood mortality and 40% of hospital attendance due to childhood illness are attributable to pneumonia, diarrhoea, measles, malaria, and malnutrition, either alone or in combination (1). Pneumonia and diarrhoea are the leading causes of under-five deaths, and the former alone is estimated to cause 2 million deaths globally each year (2). There is a need for appropriate guidelines for community-based diagnosis, intervention, and treatment for pneumonia, for incorporation into primary healthcare systems to reduce childhood mortality and morbidity (3).

The WHO guidelines for diagnosis of pneumonia in children are based on history of respiratory symptomatology, particularly cough, age-specific respiration rates, and the presence of chest in-drawing (inward movement of the bony structure of the lower chest-wall with inspiration) (4-6). Tachypnoea (increased respiration rate) and chest in-drawing are usually present in pneumonia due to decreased lung elasticity and are often accompanied with hypoxia (7). A preliminary diagnosis based on age-specific tachypnoea has been found to have both sensitivity and specificity of approximately 80% in detecting moderate and severe pneumonia in children with cough or breathing difficulties and has been used for many years as the basis of the current WHO guidelines in the treatment for pneumonia in primary-care facilities (8). A visual measurement of respiration rate over one full minute, as recommended by WHO, has been validated as accurate and consistent in pneumonia-affected children younger than five years (9). However, the combination of a chest x-ray and clinical findings observed by an experienced clinician is the accepted gold standard for diagnosis of pneumonia in primary-care settings (10).

A diagnosis of pneumonia in children with dehydrating diarrhoea is more difficult to make due to the associated metabolic acidosis which also causes tachypnoea, particularly in infants and young children (11,12). The tachypnoea in acidosis is caused by production of carbon dioxide by the blood buffer system, which needs to be excreted via the lungs but it does not reduce lung compliance as in pneumonia (13). The combination of diarrhoea and pneumonia is associated with increased case fatality (14). Although diarrhoea is a frequent diagnosis in developing countries, limited studies have examined the impact of diarrhoeal disease on the clinical diagnosis of pneumonia in a primary-care setting.

The aim of this study was to assess the effect of symptomatology of dehydrating diarrhoea on the diagnosis of pneumonia and re-evaluate the WHO diagnostic guidelines.

## MATERIALS AND METHODS

This prospective, facility-based observational study was conducted at the Dhaka Hospital of icddr,b, Dhaka, Bangladesh, from March 1999 to April 2001. Children of either sex, aged 2-59 months, attending the hospital due to acute dehydrating diarrhoea, who additionally had cough and/or difficult breathing, were eligible for the study. Those with a history of severe malnutrition, chronic respiratory, cardiac, renal or central nervous system disorders and also those with a history of cough for more than 14 days were excluded as these conditions can cause an overlap of symptoms and require other treatments. Written informed consent was obtained from the parents or guardians attending each of the participating children.

Baseline characteristics, including the assessment of dehydration, were recorded before initiating rehydration therapy (15). For this study, history of diarrhoea, fever, cough, fast breathing, breathing difficulty, and their duration was also obtained from the primary caregiver. The weight, height, and the rectal temperature of the child were measured at the time of enrollment. The investigator also observed respiratory symptoms while the child was at rest. The WHO-guided diagnosis of pneumonia was made based on age-specific respiration rate cutoffs (8). This was termed ‘WHO diagnosis 1’.

At enrollment, the investigator made his initial diagnosis of pneumonia based on history and clinical features. This was designated as ‘Investigator's diagnosis 1’.

Arterial oxygen saturation was measured using a portable pulse oximeter (Model N-10, Nellcor Inc. Hayward, California, USA), and a chest x-ray was also done. Peripheral blood white cell count, haematocrit, serum electrolyte, and creatinine concentrations were measured. Serum TCO_2_ of less than 17 mmol/L was taken as acidosis in these children (16).

The investigator re-assessed the patients six hours after the initiation of rehydration therapy, reviewed the initial chest x-ray and made a combined clinical and x-ray diagnosis of either pneumonia or no pneumonia. This was designated as ‘Investigator's diagnosis 2’ and was used as the ‘gold standard’ diagnosis of pneumonia against which other diagnoses were compared. At the same time, the diagnosis of pneumonia was repeated using the WHO guidelines based on respiration rates and was designated as ‘WHO diagnosis 2’.

### Sample-size

Our preliminary studies (unpublished observations) indicated that approximately 25% of the children with diarrhoea and cough or difficult breathing, attending the Dhaka Hospital, will have radiologically-confirmed pneumonia, and 75% will not. Therefore, we needed to examine at least 56 children with cough or breathing difficulty to get 42 children without radiologically-confirmed pneumonia. To include an approximately equal number with radiologically-confirmed pneumonia, we aimed to enroll 200 children in total.

SPSS (version 10.0 for Windows) and Epi Info (version 2000) were used for analyses of data. Continuous data were summarized by descriptive statistics and compared using the paired sample *t*-test. Sensitivity, specificity, and positive and negative predictive values were calculated. Kappa statistic (k) was calculated to assess agreement between diagnoses, using the same method at different time points (17).

### Ethical approval

The study was approved by the Research Review Committee and the Ethical Review Committee of icddr,b.

## RESULTS

We enrolled 204 children in the study but 12 were subsequently excluded for various reasons. Thus, 152 children in the 2-11 months and 40 children in the 12-59 months age-groups were available for analysis.

Table 1 describes admission characteristics of the study children. All children had a history of cough and documented fever (rectal temperature of >38.4 ºC).

The changes at enrollment and after rehydration are recorded in Table 2. Respiration rate dropped significantly 6 hours after admission in patients of both age-groups (Table 2) with correction of dehydration and metabolic acidosis (p<0.001). Consequently, the WHO diagnosis 1 and 2 changed substantially after rehydration (from 96% to 71%) but the clinical diagnosis (Investigator's diagnosis 1 and 2) did not change significantly (89% to 85%). Statistically, the agreement based on kappa score was better for Investigator's diagnosis than the agreement of the “WHO diagnosis.” The agreement for the WHO-guided diagnoses and Investigator's diagnoses of pneumonia at admission and after rehydration at 6 hours were k 0.07 and k 0.71 respectively. The change in metabolic acidosis were significant [120/192 (62.5%) on admission vs 87/192 (45.3%) at 48 hours; p<0.001].

The clinician made radiological diagnosis of pneumonia in 155 (80.7%) enrolled children at 6 hours after the initial rehydration and in 163 (84.9%) children at 48 hours. The specificity of the “WHO diagnosis” of pneumonia was very low at enrollment (6.9%), which markedly elevated after rehydration (65.5%). Even with this increase, it remained well below the specificity of 80% for the diagnosis of pneumonia based on the set criteria in the WHO guidelines. The sensitivity of the WHO guidelines fell from 96.9% to 77.9% after rehydration. The specificity of the Investigator's diagnosis 1 at enrollment was 65.5%, and sensitivity was 98.2% (Table 3).

## DISCUSSION

The study highlights the drawbacks of simplified guidelines, based on elevated respiration rates in diagnosing pneumonia in countries, such as Bangladesh where dehydrating diarrhoea is endemic. The WHO algorithm for the diagnosis of pneumonia was developed without consideration of dehydration and acidosis, which can modify the respiration rate (6). Our study is the first to assess the role of respiration rate as a predictor in diagnosing pneumonia in children with dehydrating diarrhoea.

The respiration rates varied between the dehydrated and rehydrated states of the children at enrollment and 6 hours later. The main limitation of our study was that we did not measure acidosis at 6 hours when children were apparently fully rehydrated. However, previous work indicates that acidosis is quickly corrected with correction of dehydration, usually within 3 hours (13). In our study, the Investigator's diagnoses may have been influenced by the presence of lung crepitation on auscultation that persisted after rehydration. This may account for the strong kappa value for agreement between the Investigator's clinical diagnosis between the two measurements.

**Table 1.** Admission characteristics of the study children

Characteristics	Age-group		All children (n=192)
	2-11 months (n=152)	12-59 months (n=40)	
Gender (Male); n (%)	101 (66)	29 (73)	130 (68)
Age (month)	7 (5,10)	24 (15,27)	8 (6,12)
Weight (kg)	6 (5,7)	8 (8,9)	6 (5,8)
Height (cm)	65 (61,69)	78 (75,85)	67 (62,73)
Cough; n (%)	152 (100)	40 (100)	192 (100)
Fever*; n (%)	149 (98)	40 (100)	189 (99)
Difficult breathing; n (%)	81 (53)	18 (45)	99 (49)
Fast breathing; n (%)	143 (94)	38 (95)	181 (94)
Unable to drink; n (%)	17 (11)	4 (10)	21 (11)
Abnormally sleepy; n (%)	24 (16)	7 (8)	31 (16)
Convulsion; n (%)	1 (0.7)	0 (0)	01 (0.5)
Duration of fever (hr)*	96 (72,120)	96 (72,138)	96 (72,120)
Duration of diarrhoea (hr)*	96 (54,144)	96 (53,162)	96 (54,144)
Duration of cough (hr)	96 (72,168)	120 (72,168)	108 (72,168)
Duration of fast breathing (hr)	48 (24,72)	48 (24,96)	48 (24,72)
Rectal temperature (°C)	38 (38,39)	38 (38,40)	38 (38,39)

*Three children of 2-11 months age-group did not have fever. Duration of fever, diarrhoea, cough, and fast breathing were reported by the primary caregiver of the children at the time of enrollment; Data are presented as median (interquartiles) unless specified otherwise

**Table 2.** Respiratory symptomatology and dehydration status of children on admission and 6 hours after initial rehydration

Signs and symptoms	Admission; n (%)	6 hours; n (%)	p value
Dehydration	192 (100)	41 (21)	
Some	172 (90)	41 (21)	
Severe	20 (10)	0 (0)	
Wheezing	16 (8)	14 (7)	0.79
Grunting respiration	24 (13)	22 (12)	0.80
Chest-wall in-drawing	158 (82)	121 (63)	<0.0001
Lung crepitations	153 (80)	151 (79)	0.82
Nasal flaring	27 (14)	13 (7)	0.03
O_2_ saturation (%)	96.6±7.7	97.6±2.7	0.044
Respiration rate >50 beats/min (2-11 m)* n (%)	119 (78.3)	31 (20.4)	<0.0001
Respiration rate >40 beats/min (12-59 m)* n (%)	38 (95)	10 (25)	<0.0001

* n=152 and 40 for 2-11 months and 12-59 months age-group respectively

**Table 3.** Comparison of diagnostic methods with ‘gold standard’ diagnosis based on chest x-ray and clinical examination after rehydration

Type of diagnosis	True positive	False positive	True negative	False negative	Sensitivity (%)	Specificity (%)
WHO diagnosis 1 (on admission)	158	27	02	05	96.9	6.9
WHO diagnosis 2 (after rehydration)	127	10	19	36	77.9	65.5
Investigator's diagnosis 1 (on admission)	160	10	19	03	98.2	65.5

The overall rate of diagnosis of pneumonia was very high, with 85% of children being diagnosed with pneumonia by x-ray and clinical examination after rehydration. This is always a problem in studies of pneumonia, where no absolute ‘gold standard’ test for diagnosis exists—chest x-rays may mislead, and lung crepitations have certainly been earlier reported in children with dehydration, who do not have pneumonia (18). However, the direction of any bias introduced by the Investigator's overdiagnosis would be towards increased specificity of the WHO guidelines against Investigator's diagnosis 2, which we regarded as the ‘gold standard’. This study did not show such increase in the specificity. Hence, the finding of greatly reduced specificity of the WHO guidelines indicates a valid observation.

We noted a very low specificity of 6.9% for the WHO diagnostic algorithm at the initial presentation of children younger than 5 years, who had dehydrating diarrhoea and cough. Based on this level of specificity, the WHO guidelines provided only marginal benefit over simply treating all children with respiratory symptoms and diarrhoea with antibiotics, without any attempt for diagnosis.

We conclude that the specificity of the WHO guidelines-based diagnosis of pneumonia is reduced from the estimated 80% to less than 10% at initial presentation in children with dehydrating diarrhoea. In hospital-based management, full clinical assessment and chest x-ray will continue to remain valuable for dehydrated children with respiratory symptomatology. Healthcare providers in the community and in facilities without such provisions could possibly be encouraged to re-assess patients after correction of acute dehydration before applying the WHO guidelines and initiating antimicrobial therapy for pneumonia in children with concomitant dehydrating diarrhoea.

## ACKNOWLEDGEMENTS

This study was funded by the United States Agency for International Development (USAID) under the Cooperative Agreement No. 388-A-00-97-00032-00. icddr,b acknowledges with gratitude the commitment of the USAID to its research efforts. We thank Mr. Humayun Kabir for preparing the case report form for the study and data-entry; Mrs. Nurer Nahar and Mrs. Mafruha Ahsan for screening of potential study children and their initial assessment; and the nurses and medical staff of the Dhaka Hospital of icddr,b for their excellent patient-care and support to the participating children in the study. Finally, we thank the study children and their parents for taking part in this study.
